# Acquisition and Neural Network Prediction of 3D Deformable Object Shape Using a Kinect and a Force-Torque Sensor †

**DOI:** 10.3390/s17051083

**Published:** 2017-05-11

**Authors:** Bilal Tawbe, Ana-Maria Cretu

**Affiliations:** Department of Computer Science and Engineering, Université du Québec en Outaouais, Gatineau, J8X 3X7 QC, Canada; ana-maria.cretu@uqo.ca

**Keywords:** deformation, force-torque sensor, Kinect, iterative closest point, neural gas, neural networks, clustering, mesh simplification, RGB-D data, 3D object modeling

## Abstract

The realistic representation of deformations is still an active area of research, especially for deformable objects whose behavior cannot be simply described in terms of elasticity parameters. This paper proposes a data-driven neural-network-based approach for capturing implicitly and predicting the deformations of an object subject to external forces. Visual data, in the form of 3D point clouds gathered by a Kinect sensor, is collected over an object while forces are exerted by means of the probing tip of a force-torque sensor. A novel approach based on neural gas fitting is proposed to describe the particularities of a deformation over the selectively simplified 3D surface of the object, without requiring knowledge of the object material. An alignment procedure, a distance-based clustering, and inspiration from stratified sampling support this process. The resulting representation is denser in the region of the deformation (an average of 96.6% perceptual similarity with the collected data in the deformed area), while still preserving the object’s overall shape (86% similarity over the entire surface) and only using on average of 40% of the number of vertices in the mesh. A series of feedforward neural networks is then trained to predict the mapping between the force parameters characterizing the interaction with the object and the change in the object shape, as captured by the fitted neural gas nodes. This series of networks allows for the prediction of the deformation of an object when subject to unknown interactions.

## 1. Introduction

The acquisition and realistic simulation of deformations, especially for soft objects whose behavior cannot be simply described in terms of elasticity parameters, is still an active area of research. Realistic and plausible deformable object models require experimental measurements acquired through physical interaction with the object in order to capture its complex behavior when subject to various forces. The measurement of the behavior of an object can be carried out based on the results of instrumented indentation tests. Such tests usually involve the monitoring of the evolution of the force (e.g., its magnitude, direction, and location) using a force-feedback sensor. The active indentation probing is usually accompanied by a visual capture of the deformed object to collect geometry data characterizing the local surface deformation. 

A classical high precision solution for collecting 3D geometry data are laser scanners. However, they are expensive and the acquisition process is often lengthy. The Kinect sensor proves to be a simple, fast, and cost-effective alternative. It is however known that, while small objects are difficult to capture with the sensor due to its relatively low resolution, it offers enough precision for measuring objects with a reasonable size, such as a ball or a sponge, as used in this work. RGB-D (red, green, blue and depth) data collected by this sensor has been successfully used for the reconstruction of complete 3D pointclouds of objects by merging data from multiple viewpoints, for both rigid [1] and non-rigid objects [2,3]. A few open-source options to integrate and stitch multiview data, such as Kinect Fusion [4], and commercial solutions, such as Skanect [5], are also available. 

Once the 3D data is acquired, various approaches can be used for modeling a deformable object such as: mass-spring models, the finite element method, viscoelastic tensor-mass models, Green functions [6], NURBS (Non-uniform rational basis splines), or surfels [1]. Most of these techniques assume that the parameters describing the object behavior (e.g., elasticity parameters) are known a priori or values for these parameters are chosen by manually tuning them until the results seem plausible. This is a subjective process and while it can be used for certain types of applications, it cannot be employed where a certain level of accuracy is expected, as an improper choice of parameters can lead to undesired behavior. 

The objective of this work is therefore to propose an original solution to the modeling and prediction of the deformation of soft objects that does not make assumptions about the material of the object and that capitalizes on computational intelligence solutions, namely on combining neural gas fitting with feedforward neural network prediction. It builds on a previously proposed solution [7] for capturing the deformed object shape using neural gas fitting. However, the modeling process is improved in this work by an alignment procedure that ensures a simpler interpretation of the deformation (e.g., simpler comparison for various angles and forces) and a similar treatment of the deformation over the surface of an object. In addition, an in-depth analysis conducted on the parameters leads to a more appropriate choice of parameters, that results in a better performance for object representation. Finally, a novel solution is proposed based on a neural network architecture to predict the deformed shape of an object when subjected to an unknown interaction.

The organization of the paper is as follows: After a general introduction on deformable object acquisition and modeling and the associated challenges in Section 2, the proposed framework for 3D deformable object modeling and prediction using a Kinect and a force-torque sensor is presented in Section 3. The obtained experimental results are presented and discussed in Section 4. The paper is concluded in Section 5.

## 2. Related Work 

Several solutions have been proposed in the literature for modeling deformable objects, including: mass-spring model [8,9], finite-element (FEM) representation [10,11,12], Green functions [6], NURBS [13], and surfels [1], just to mention a few. Most of these techniques assume that the parameters describing the object behavior (e.g., elasticity parameters) are known a priori or that values for these parameters are chosen by manually tuning them until the results seem plausible. This is a subjective process and while it can be used for certain types of applications, it cannot be employed where accuracy is expected, as an improper choice of parameters can lead to undesired behavior. To overcome this problem, several researchers propose recuperating the parameters based on a comparison between the real and simulated object subject to interactions. Zaidi et al. [8] modeled an object in an interaction with a robot hand as a non-linear isotropic mass-spring system, but do not capture visual data to build the model. Object deformations are computed based on tracking the node positions and solving the dynamic equations of Newton’s second law. Elbrechter et al. [9] modeled a piece of paper in an interaction with a robot hand as a 2D grid of nodes connected by links that specify the bending constraints and a stiffness coefficient. The parameters of the model are tuned manually. The authors of [11] tracked deformable objects from a sequence of point clouds by identifying the correspondence between the points in the cloud and a model of the object composed of a collection of linked rigid particles, governed by dynamical equations. An expectation-minimization algorithm found the most probable node positions for the model given the measurements. Experimentation is performed in a controlled environment, against a green background, which limits its applicability to real conditions. Choi et al. [14] tracked the global position of moving deformable balls, painted in red against a blue background, in a video stream and adjusted the elasticity parameters of a mass-spring model of the ball by optimizing the differences with the real object. Petit et al. [12] tracked 3D elastic objects in RGB-D data. A rigid transformation from the point cloud to a linear tetrahedral FEM model representing the object was estimated based on the iterative closest point (ICP) algorithm. Linear elastic forces exerted on vertices are computed from the point cloud to the mesh based on closest point correspondence and the mechanical equations are solved numerically to simulate the deformed mesh. Their work focuses solely on elastic objects. In [15], the stiffness properties of mass-spring models were estimated by applying force constraints at different locations on the object, recording the resulting displacements and comparing them between the reference and the estimated model using particle filters. In a similar way, the authors of [16] presented a genetic-based solution to identify stiffness properties of mass-spring-systems by using a linear FEM model as a reference. While the method seems to support isotropic and anisotropic reference models, only simulated results are presented. In order to compute the elasticity parameters, Frank et al. [10] minimized the difference between the real object in interaction with a force-torque sensor and captured it visually as a pointcloud and the simulated object in the form of a linear FEM model. However, their method only works on homogeneous and isotropic materials. These methods justify the interest in the development of new methods that do not make assumptions on the material of the object, such as the one proposed in this paper.

## 3. Proposed Framework for 3D Deformable Object Modeling and Prediction

**Figure 1:**
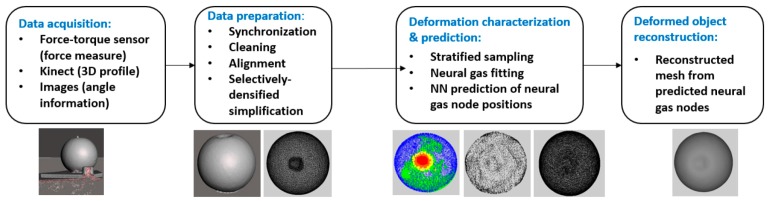
Proposed framework for deformation data acquisition, modeling, and prediction.

Figure 1 summarizes the main steps of the proposed solution for data acquisition, modeling, and prediction for 3D deformable objects.

In order to collect data on the object deformation, forces are exerted by physical interaction with the probing tip of a force-torque sensor. During this interaction, the force magnitude is monitored as well as the angle of the probing tip with respect to the object’s surface. At the same time, the object surface deformation is collected in the form of a pointcloud using a Kinect sensor and as a commercially-available solution for the integration of RGB-D data pointclouds into a coherent 3D object model [5]. Based on the acquired deformation and measured force parameters, the proposed solution captures implicitly the object behavior by employing computational intelligence techniques. The particularities of the deformation are initially identified over the aligned 3D surface of the object using knowledge about the interaction point with the probing tip and a representation of the deformed instance of an object is constructed by capitalizing on a stratified sampling procedure based on the deformation depth, followed by a neural gas fitting. A feedforward neural-network solution then learns to predict a mapping between the measured parameters characterizing the interaction with the object (force magnitude, angle, etc.) and the change in the object shape (captured implicitly by neural gas nodes) due to the interaction. The inherent parallel structure and the potential for hardware implementation of neural networks also provide an efficient way to ensure real-time prediction while still using real, raw noisy data, as opposed to the use of different approximations and assumptions on the object material, found nowadays in the literature. Therefore, the proposed solution avoids recuperating elasticity parameters which cannot be precisely and accurately identified for certain materials such as sponge or rubber, as those used in the context of this work. This means that the proposed solution does not need to make assumptions on the material, such as its homogeneity or isotropy, assumptions that are often encountered in the literature. Due to this property, the method can be used as is to characterize any object material or material deformation stages, including elastic, elasto-plastic, and even rigid objects.

### 3.1. Data Acquisition

**Figure 2:**
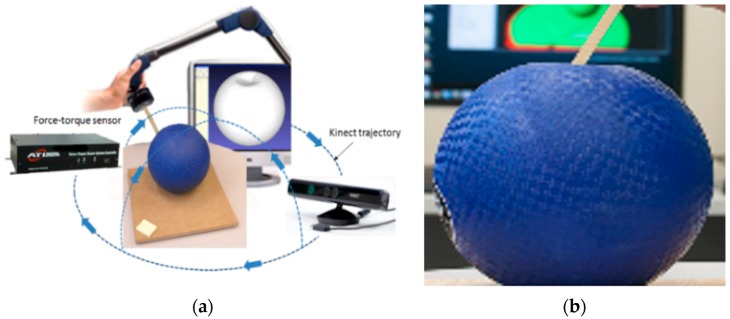
(**a**) Experimental platform for collecting data on deformable object deformation behavior and (**b**) example of an image for angle data calculation.

The experimental platform, illustrated in Figure 2a, contains a Kinect sensor and an ATI force-torque sensor equipped with a probing tip. The Kinect is employed to collect 3D pointclouds representing deformable objects by turning the sensor around the object of interest following the trajectory marked by blue arrows in Figure 2a and integrating the partial collected pointclouds using a commercially available software [5] while a constant force is applied on the object using the force-torque sensor. Three-dimensional data are collected over the surface of the object with various force magnitudes at various angles of the force-torque sensor with respect to the normal plane on the surface. The interaction parameters with the force-torque sensor are recuperated via a serial port. A Tera Term [17] script is used to initiate the data transfer and collect in real-time the measured force magnitude over the *x*, *y*, and *z* axes sent by the sensor. The force magnitude *F* at point *P* at the moment *t* can then computed as the norm of the three orthogonal force components along the *x*-, *y*-, and *z*-axes of the force/torque sensor’s reference frame returned by the sensor:

(1) $$F_{P}=\sqrt{F_{x}^{2}+F_{y}^{2}+F_{z}^{2}}$$

It is important to mention that the raw data collected need to be corrected due to the fact that a probing tip is attached to the sensor and its weight has an influence on the measured force components. At the same time with the force data acquisition, images of the setup (an example is shown in Figure 2b) are collected using a camera during each measurement step, each for 30 s, in order to recuperate with the help of image processing software the angle between the probing tip and the object surface. In particular, the probing tip is extracted from each image based on color information and morphological operations are applied to eliminate the noise. The resulting probing tip is thinned (i.e., the image is simplified into a topologically equivalent image in which the skeleton of the probing tip is obtained by mathematical morphological operations) to enable the computation of the angle with respect to the normal plane on the object surface. The angle is computed between the obtained skeleton representing the probing tip and the direction parallel to the top-bottom side of the image.

### 3.2. Data Preparation

The data preparation procedure contains four steps, namely: data synchronization, data cleaning, data alignment, and data simplification. These are described and justified in the following sections.

#### 3.2.1. Data Synchronization

Because of the different sampling rates of the force-torque sensor, of the angle information collection, and of the 3D data acquisition, a synchronization process is required in order to associate the correct surface deformation with the corresponding angle and force magnitude measurements. This is achieved by calculating the mean of all measurements collected over the time it takes for the 3D model to be acquired (e.g., in the interval *t*_1_ − *t*_2_), namely: (1) the mean of the measured force magnitude values returned by the force-torque sensor for each component of the force along the *x*, *y*, *z* axes, respectively:

(2) $$F_{aj}=\sum_{t=t_{1}}^{t_{2}}F_{j}/n$$

where *j* = {*x*, *y*, *z*} and *n* is the number of readings returned by the sensors in the interval *t*_1_ − *t*_2_ and (2) the mean of the force angle computed over the series of images:

(3) $$a_{Pa}=\sum_{t=t_{1}}^{t_{2}}a_{P}/m$$

where *m* is the number of images captured (similar to the ones in Figure 2b) in the interval *t*_1_ − *t*_2_. The deformed object model is therefore considered to be the result of the application of a force with a magnitude equal to the computed average magnitude along the *x*, *y*, *z* axes respectively: $F_{ax}$, $F_{ay}$, and $F_{az}\;\left(\mathrm{Equation}\;\left(2\right)\right)$, and $F_{Pa}=\sqrt{F_{ax}^{2}+F_{ay}^{2}+F_{az}^{2}}$, which is applied at an angle equal to the calculated average angle value, $a_{Pa}$ (Equation (3)).

#### 3.2.2. Data Cleaning

**Figure 3:**
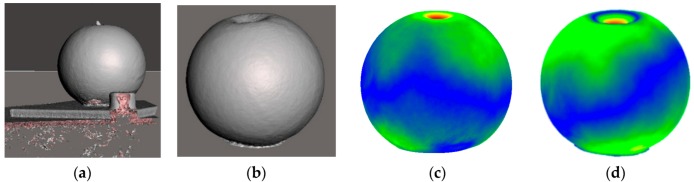
(**a**) Raw data collected; (**b**) preprocessed data; and (**c**,**d**) deformation distributions with respect to the non-deformed object when applying a light and a strong force, respectively; regions in blue are not deformed, and the deformation is getting stronger from green to red.

The 3D data collected using the Kinect sensor contains undesired elements, such as the table on which the object is placed, the fixed landmarks required by the software to merge data from multiple viewpoints, and the probing tip, as can be noticed in Figure 3a. These are eliminated in part both automatically (e.g., table and landmarks) and manually (e.g., the probing tip that is acquired as part of the object). Due to the fact that during experimentation the object is placed on a table (Figure 2), an efficient way to remove most of the background is to locate the planar surface representing the table and extract it from the RGB-D data. The flat surface identification is viewed as a plane-fitting problem which is resolved with the random sample consensus (RANSAC) algorithm [18]. Once the best plane model is obtained by the algorithm, the flat surface and the background information, represented by the inliers of this model, is removed from the point cloud. To automatically remove the landmark, only the largest component remaining in the scene, which is the object, is considered for further processing. This is a reasonable assumption, because the landmark used during experimentation is relatively small with respect to the size of the object (shown in white, in the bottom left corner of Figure 2a). The holes in the object around the probing tip resulting from its removal are filled using Meshmixer [19], a mesh processing software, in order to obtain clean representations of the deformable object. 

Figure 3b shows an instance of cleaned 3D data, while Figure 3c,d encode in color the regions that are displaced during an interaction with a soft and a strong force, respectively. By comparing the initial non-deformed object with a deformed object using CloudCompare [20], one can notice in blue the regions that are not deformed, while for the areas going from green to yellow, orange, and red, the deformation is getting stronger. Figure 3c,d show that, in fact, not only the area around the probing tip is affected by the interaction, but a much larger area of the object. Such behavior is extremely difficult, if not impossible, to capture by existing 3D modeling techniques, such as mass-spring models or FEM.

#### 3.2.3. Data Simplification

**Figure 4:**
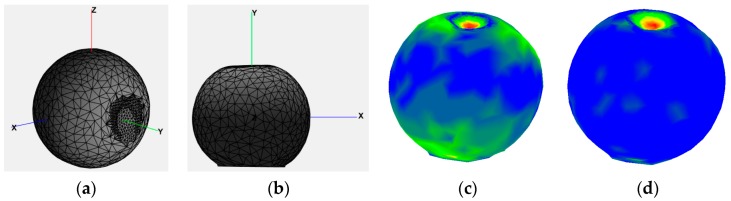
(**a**) Aligned model with *x*, *y*, *z* axes, (**b**) *x*, *y* view; and difference between a model when a light force is applied on the top at an angle of 75° with respect to the y axis and the reference model (**c**) before ICP and (**d**) after ICP alignment.

In order to better characterize the deformation around the probing point, instead of using the entire collected pointcloud, **M**, we first constructed a selectively-densified mesh, **M_s_** (of which an example is shown Figure 4a), in which the area around the point of interaction between the probing tip and the object surface is preserved at higher resolution, while the other areas are simplified. This ensures that the small deformed area around the probing tip has a higher density of points with respect to the rest of the object’s surface. In order to achieve this, similar to the approach proposed in [21], the QSlim [22] algorithm was adapted to only simplify points that are not the interaction point with the probing tip and its *n*-degree immediate neighbors. Algorithm 1 summarizes the proposed object simplification process. For experimentation, we chose 12-degree neighbors for the ball and 8-degree neighbors for the cube and the sponge, respectively. These were selected in such a way that the entire deformation area is covered in all deformed instances of the object within this neighborhood. Details on the selection of 12 and 8 neighbors, respectively, are provided in Section 4. This process allows us to define an equal number of faces for all the instances of a deformed object, as the number of faces is a parameter to be provided in the simplification process. It therefore ensures a uniform representation of all deformed instances of an object.
**Algorithm** **1.** Object simplification using QSlim.Input: **M** = cleaned acquired meshOutput: **M_s_** = simplified mesh (denser around probing point)Step 1: Identify the point of intersection **I** of the probing tip with the object mesh: **I** = [*X*, *Y*, *Z*], **I**
∈
**M**Step 2: // simplification for a mesh **M** to obtain selectively-densified mesh **Ms** Calculate **IN** = the *n*-nearest neighborhood of point **I** in **M** // identify only edges that do not belong to **IN**  Initialize **Ms** = {*e(u,v)* | *e(u,v)*
∈
**M**−
**IN**} // apply QSlim  Compute quadric error at each vertex of **M_s_**  Determine contraction cost at each edge *e(u,v)* in **M_s_**  Create ordered list of edges based on costStep 3:  Remove the edge *e(u,v)* with lowest cost  Use quadric error to choose the optimal contraction target  Contract *u* and *v* and recalculate costs for the adjacent edges in **Ms**While desired number of faces not reached, repeat Step 3

#### 3.2.4. Data Alignment

In order to ensure a simpler interpretation of the deformation (e.g., comparison for various angles and forces) and a similar treatment of the deformation over the surface of an object, the objects are realigned such that the deformed zone is situated on the top of the object. This operation is especially useful for symmetrical objects, such as the ball, but improves the interpretation of the deformed zone for all types of objects. The alignment procedure is executed in two stages: In the first stage, the object is rotated such that the contact point with the force-torque tip is aligned along the *y* axis. The *z* axis points in this case towards the user, *y* is the up-down direction and *x* is the left-right direction, as shown from two viewpoints in Figure 4a,b, while Figure 4c shows an example of the aligned model, **M_a_axis_**, when compared to a reference model, **M_r_**. The latter is selected to be the one in which the strongest force (with respect to the capabilities of the sensor used, e.g., 10 N) is applied in the normal direction on the top of the object, but other deformed models could be used as well. The reason to choose a reference model that is already deformed instead of its non-deformed initial state is to ensure a better alignment of the two models around the deformation zone. 

In a second stage, a fine alignment is ensured between the reference model, **M_r_** and each simplified and aligned model, **M_a_axis_**, using the iterative closest point (ICP) algorithm [23]. In order to ensure good results, the latter requires that the two models are roughly aligned, from which stems the necessity of the initial axis alignment in the first stage. An example of color-encoded differences obtained using Cloud Compare between the non-deformed object model and a model in which a light force (4 N) is applied on the top at an angle of 75 degrees with respect to the *y* axis is shown after the axis alignment, but prior to ICP alignment in Figure 4c and after ICP alignment in Figure 4d. One can notice that after the ICP alignment, the differences focus mainly on the deformed zone and not over the entire surface of the ball (e.g., there are more blue areas around the ball surface, showing a better fitting). The combined transformation (translation and rotation) matrix returned by ICP is saved in order to allow the repositioning of the deformed zone at its correct place over the surface of the object.

This step is summarized in Algorithm 2.
**Algorithm**
**2.** Object alignment.Input: **M_s_** = selectively densified mesh   **M_r_** = reference meshOutput: **M_a_** = aligned mesh with reference objectStep 1:  // Rough axis alignment  Align **M_s_** along *y* axis to obtain **M_a_axis_**Step 2:  // ICP refinement  For each point **P**
∈
**M_a_axis_** find the closest point **P_r_**
∈
**M_r_**   Calculate mean-squared error between **P** and **P_r_**   Estimate the combination of translation and rotation to ensure best alignment between **P** and **P_r_** based on the   computed error  Transform the source points using the transformations to obtain **M_a_**  Store the transformation matrix.While error larger than threshold repeat Step 2 

### 3.3. Object Deformation Characterization

The object deformation characterization procedure comprises three steps, namely: data sampling, neural gas network fitting, and neural network prediction of neural gas nodes.

#### 3.3.1. Sampling

In order to enable the characterization and prediction of the object deformation, it is necessary to reduce its complexity. In this paper, this issue is addressed in part by only using a lower number of points from the selectively-densified mesh, using inspiration from stratified sampling and in part by the neural gas fitting procedure described in the next section. 

Stratified sampling is a technique that generates samples by subdividing the sampling domain into non-overlapping partitions (clusters) and by sampling independently from each partition. In particular, the ICP aligned mesh, **M_a_**, is clustered according to the distance to the initial non-deformed mesh. In order to detect the number of clusters to use, the normalized interval between the deformed mesh and each instance of the object under study is gradually split in an increasing number of equal intervals (number of clusters to be used) and the points in the deformed area (probing tip and its immediate neighbors) are compared with the cluster situated at the largest distance. This cluster should be correlated with the deformed area and it is desired that a high number of deformed zones are situated within it. During testing, the number of points of the deformed zone in the largest cluster is monitored, and the process is stopped once the largest number of points in the deformed zone is found in this cluster. 

Once the number of clusters is identified, points are sampled randomly but in various proportions from each cluster to identify the adequate amount of data to be used. The proportions were varied during experimentation, by taking into consideration the fact that a good representation is desired specifically in the deformed area and therefore more samples are desired for regions in which the deformation is larger. In the context of this work, we gradually decreased the percentage of points sampled from the farthest cluster to the closest cluster representing the deformed zone, namely 90% from the farthest cluster (deformed area), 80% from the 2nd, 70% from the 3rd, and so on. 

#### 3.3.2. Neural Gas Network Fitting

Once the data is sampled, the combination of sampled data, resulting from the various percentages as described above, is fitted with a neural gas network. The choice of this network is justified by the fact that it converges quickly, reaches a lower distortion error, and better captures finer details than other self-organizing architectures [24,25]. The latter is important in order to ensure that fine differences (i.e., fine green peaks over the surface of the object in Figure 3d) can be captured in the model. The input point cloud for the neural gas contains in this case the *X*, *Y*, and *Z* coordinates of the points sampled from the clusters. 

While the fact that the neural gas map size has to be chosen a priori is in general a drawback, it is an advantage in the context of the current work as it maintains a constant size for the representation of a deformed object regardless of the initial size of the object collected by the Kinect. While the entire description of neural gas is available in [26], and its adapted version for 3D pointclouds in [25], the neural gas fitting process used in this paper is summarized in Algorithm 3.
**Algorithm**
**3.** Neural gas fitting.Input:  **P_si_** = stratified sampled points from clusters C_1_ to C_5_Output: **NG** = fitted neural gas network Initialize neural gas network **NG** and set parameters:      O = {*P_1_*, … *P_i_*, …, *P_NS_*}, *P_i_* = [*X_i_*, *Y_i_*, *Z_i_*] ∈
**P_si_**     $\alpha \left(t\right)=\alpha_{o}{\left(\alpha_{T}/\alpha_{o}\right)}^{t/T}$; $\lambda \left(t\right)=\lambda_{o}{\left(\lambda_{T}/\lambda_{o}\right)}^{t/T}$;  Initialize the weights $w_{i}$ with small random values; // apply neural gas adaptation over **O** to obtain the neural gas map: **NG_i_** = {*P_1_*, …, *P_j_*, …, *P_N_*}| *P_j_* = [*X_j_*, *Y_j_*, *Z_j_*], *j* = 1, …, N;  while *t* < *T*
    Select a point **P_o_** at random from **O**
    for *j* = 1:1:N    Sort weights according to the distance to **P_o_**    Update weights $w_{j}\left(t+1\right)=w_{j}\left(t\right)+\alpha \left(t\right)h_{\lambda }\left(k_{j}\left(x,w_{j}\right)\right)\left[x\left(t\right)-w_{j}\left(t\right)\right]$; where $h_{\lambda }\left(k_{j}\left(x,w_{j}\right)\right)=exp\left(-k_{j}\left(x,w_{j}\right)/\lambda \left(t\right)\right)$    Reduce learning rate α
   end  end

#### 3.3.3. Neural Network Prediction of Neural Gas Nodes Position 

**Figure 5:**
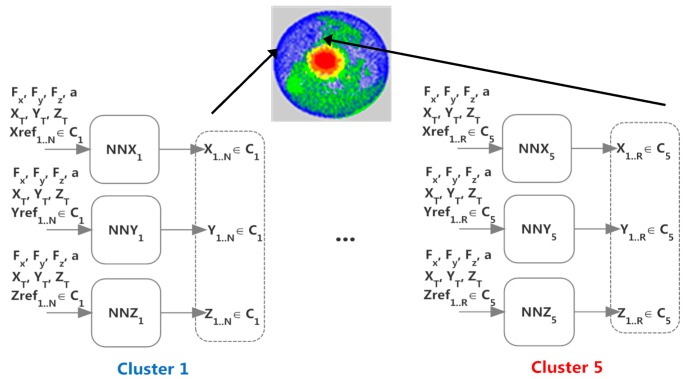
Feedforward architecture for neural gas node prediction.

Once a neural gas representation is built for each instance of a deformable object, a series of two-layer feedforward networks, three per each cluster of an object, denoted *NNX*, *NNY*, and *NNZ* in Figure 5, and corresponding to the *X*, *Y*, and *Z* coordinates, respectively, is trained to map the complex relationship between the force and angle data and the corresponding position of neural gas nodes over each cluster. 

Each network takes as inputs the force components $F_{x}$, $F_{y}$, and $F_{z}$, the angle a, the coordinates *X_T_*, *Y_T_*, and *Z_T_* of the point of intersection **I** of the object with the probing tip, and a series of *X*, *Y*, and *Z* coordinates. In an attempt to simply the complex learning scenario, the network learns to map between the neural gas nodes representing the reference model **M_r_** of an object and any deformed stage of the object. Therefore the *X*, *Y*, and *Z* coordinates in the input will correspond to neural gas nodes representing the reference model **M_r_** and are denoted *X_ref_*, *Y_ref_*, *Z_ref_*, respectively, in Figure 5. Each network contains 20 sigmoid hidden neurons and linear output neurons and uses the scaled conjugate gradient backpropagation for training. Each is trained with the measured data using 90% of the data for training, 5% for validation, and 5% for testing.

#### 3.3.4. Deformed Object Reconstruction 

Once the series of neural networks is trained, they can be used conjunctly to predict the position of neural gas nodes for force magnitudes and angles that were not measured, and therefore data with which the network was not trained. *X*, *Y*, *Z* coordinates of points predicted for each cluster by the series of the neural networks in Section 3.3.3 are assembled together to provide an estimate of the entire object shape. In order to reconstruct the object, one solution would be to construct a mesh directly over the predicted points, using for example a Poisson surface reconstruction [27]. However, this is not an optimal solution in this case, due to the fact that we concentrated the effort in the deformed area and sampled much less points for the rest of the object. If the neural gas nodes are used as predicted, the quality of the model will be significantly deteriorated over most of the surface of the resulting mesh. 

A solution to deal with this problem is to use the adapted data simplification algorithm that we presented in Section 3.2.3, but to constrain the number of triangles in the mesh to be equal to the average number of faces in the reference mesh model, **M_s_**, representing the various deformation instances of an object. In this case, instead of performing a simplification operation, the adapted algorithm will force the redistribution of faces in those areas in which changes occurred as a result of the deformation. 

### 3.4. Model Validation

To evaluate the results of the object representation and prediction using the proposed approach, as well as allow the selection of parameters, the similarity of the reconstructed model obtained in Section 3.3.4 is compared to the cleaned object model (collected by the Kinect) qualitatively (visually) and quantitatively (error calculation). 

The difference in deformation between the acquired data belonging to a real object and its model is visualized using CloudCompare [20]. Using the obtained, simplified representation as one model and the original full-resolution mesh of the object as another model, the difference between them will highlight the areas most affected by errors. For a quantitative measure of errors, the Metro tool [28] allows, in a similar manner, to compare two meshes (e.g., the original, full-resolution mesh, and its simplified version) based on the computation of a point-surface distance and returns the maximum and mean distance, as well as the variance (RMS). The lower this error is, the better the quality of the simplified object is. Since our interest is in improving the perceptual quality of the models, another measure of perceptual error is also employed. It is based on the structural similarity metric (SSIM). The latter was proposed based on the observation that human visual perception is highly adapted to extract structural information in a scene [29]. The inverse of this metric can be employed as an error measure, because a lower similarity between the simplified mesh and the initial mesh implies a higher error. Because this measure is meant to be applied on images, in order to compute it, images are captured over the simplified models of objects from various viewpoints and are compared with the images of the initial, non-simplified object from the same viewpoints. In the current case, the object is turned around the *y* axis for 360°, 15° at a time, and images are collected over the resulting 25 viewpoints. This measure is reported for each deformed instance of an object as an average over these viewpoints. 

## 4. Experimental Results

**Figure 6:**
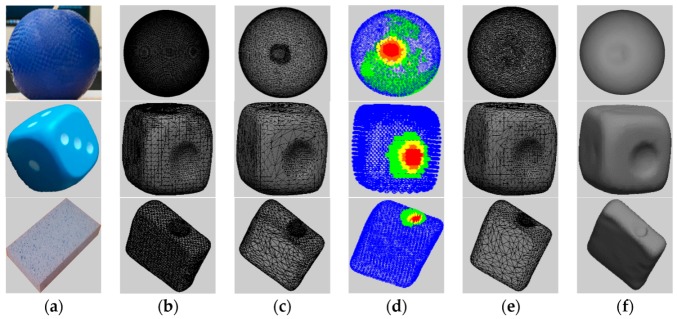
(**a**) Object model; (**b**) initial object mesh; (**c**) mesh with higher density in the deformed area; (**d**) stratified sampled data for neural-gas mapping; (**e**) neural-gas-tuned simplification; and (**f**) neural-gas-tuned simplified object.

The proposed solution for modeling and predicting deformable object shapes is validated in this paper by using a soft rubber ball, a rubber-coated foam cube, and a foam sponge (Figure 6a). Starting from the cleaned mesh, instead of using the entire collected pointcloud (Figure 6b), a selectively-densified mesh (Figure 6c) is first constructed, in which the area around the point of interaction between the probing tip and the object surface is preserved at higher resolution, while the other areas are simplified. This is achieved by adapting the QSlim [22] algorithm to only simplify points that are not the interaction point with the probing tip and its immediate neighbors. 

**Figure 7:**
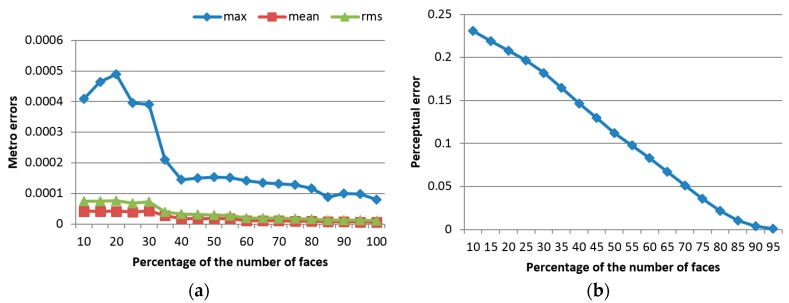

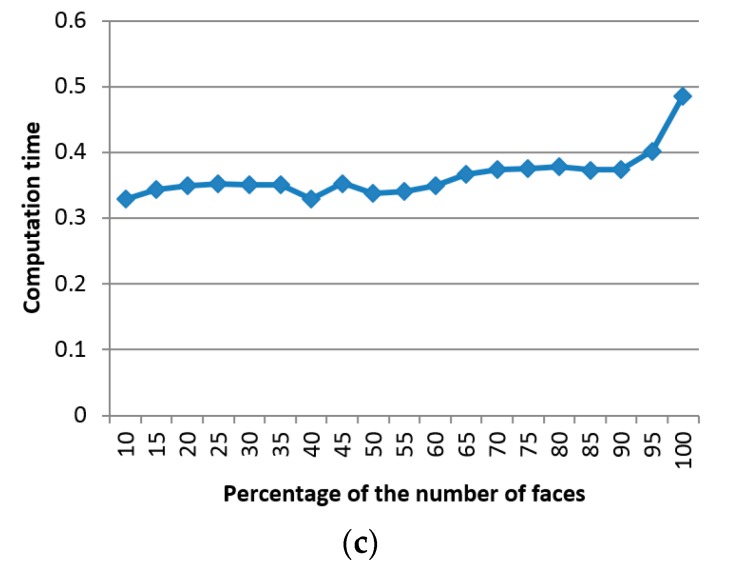
Evolution of: (**a**) Metro error; (**b**) perceptual error; and (**c**) computation time with the number of faces used for data simplification.

This simplification process ensures a uniform representation of the object by defining an equal number of faces, representing 40% of the faces in the initial model for all the instances of a deformed object. This 40% is chosen by monitoring the evolution of the errors and of the computation time for an increasing percentage (from 10% to 100%) and finding the best compromise between the two. Figure 7 shows the evolution of the error measures detailed in Section 3.4, namely of the Metro errors (Figure 7a), the perceptual error (Figure 7b), and of the computation time (Figure 7c), calculated as an average over the entire surface of the three objects under study, with the percentage of faces used for simplification. One can notice that, as expected, the error decreases with an increase in the number of faces. At the same time, the computation time increases slightly with a larger number of faces, and therefore the best compromise is found between these two opposing criteria. The abrupt descent in Metro error and the relatively low computation time around 40% of the number of faces guided our choice for the selection of this parameter. 

A second parameter that affects the simplification algorithm is the number of immediate neighbors that are preserved around the probing tip. In [7], we have used 12 immediate neighbors for all the objects. In the current work, the 12-degree immediate neighbors are preserved for the rubber ball and 8-degree immediate neighbors for the foam sponge and the cube. Only values within this range (8 to 12) are tested, because they allow us to correctly capture the entire deformed area over the 3D point clouds collected. Within this range, the number of neighbors is chosen by selecting the model that results in the lowest perceptual error (or the highest perceptual similarity) with respect to the original, non-simplified mesh. Table 1 shows the evolution of the perceptual errors with the size of the neighborhood (*n*) around the probing tip and justifies the choice of the neighborhood size based on the highest similarity.

**Figure 8:**
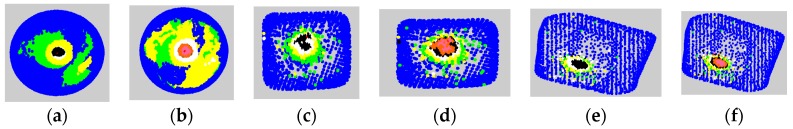
Clusters for ball: (**a**) 5 clusters; (**b**) 7 clusters; for cube: (**c**) 5 clusters and (**d**) 7 clusters; and for sponge: (**e**) 5 clusters and (**f**) 7 clusters.

The results for the selective simplification around the probing tip with the above detected values for the parameters are shown for the three test objects in Figure 6c. After having obtained the selectively-densified mesh, a stratified sampling technique, as detailed in Section 3.3.1, is employed to only retain a subset of data for neural-gas tuning. In particular, the mesh is initially clustered according to the distance with respect to the initial non-deformed mesh. It is desired that the highest possible number of points from the deformed zone is situated in the farthest cluster. In our previous work [7], during testing, the number of points of the deformed zone in this cluster was monitored, and the process was stopped once the largest number of points in the deformed zone was found in this cluster. A number of five clusters was identified to ensure the best results. In this work, a more thorough analysis led to a better definition of the area around the deformation zone by increasing the number of clusters and regrouping the ones at the largest distance to cover the entire deformation area. In this case, an equal number of five clusters were used, but by identifying seven clusters and regrouping the three farthest together. Figure 8 shows the difference, for each of the test objects, between the use of five and seven clusters. One can notice that in all three cases, the use of seven clusters and the combination of clusters at the farthest distance more accurately represents the deformation area. For example, for the case of the ball, the combination of the red, fuschia, and orange clusters in Figure 8b results in a more accurate representation of the deformation area than the black cluster in Figure 8a. The same conclusion can be reached by comparing Figure 8c,d and Figure 8e,f, respectively.

Figure 6d shows the clusters, with red points belonging to the farthest cluster from the initial object (deformed zone) and with orange, yellow, green, and blue points being increasingly closer with respect to the initial mesh (blue = perfect match), for the combination of 90% from the farthest (red) cluster, 80%, 70%, and 60%, respectively, from the 2nd, 3rd, and 4th cluster, and 50% from the closest distanced cluster points (blue). While a higher percentage might achieve better results, it will also result in a higher computation time.

**Figure 9:**
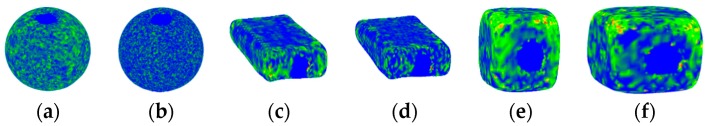
Color-coded results for the ball, sponge, and cube with respect to the initial full-resolution model: (**a**) selectively-densified mesh around the probing point for a ball for $F_{Pa}$ = 4.5 N, $a_{Pa}$ = 10°; (**b**) final mesh for a ball for $F_{Pa}$ = 4.5 N, $a_{Pa}$ = 10°; (**c**) selectively-densified mesh around the probing point for a sponge for $F_{Pa}$ = 3.7 N, $a_{Pa}$ = 49°; (**d**) final mesh for a sponge for $F_{Pa}$ = 3.7 N, $a_{Pa}$ = 49°; (**e**) selectively-densified mesh around the probing point for a cube for $F_{Pa}$ = 5 N, $a_{Pa}$ = 85°; and (**f**) final mesh for a cube for $F_{Pa}$ = 5 N, $a_{Pa}$ = 85°.

The stratified sampling is not sufficient, as the fine differences around the deformed zone and over the object surface (Figure 3c,d) might not be appropriately represented, which is the reason why a tuning of the selectively densified mesh is also executed using a neural gas network. A neural gas network is adapted, as explained in Section 3.3.2, for 20 iterations. It uses a number of neurons in the map equal to the number of points sampled from the different clusters and a number of faces equal to the average of the number of faces over the selectively densified meshes. The object model is then constructed using the solution proposed in Section 3.3.4. Figure 9 presents the color-coded comparison with the initial full resolution object obtained using CloudCompare for each of the three objects after the construction of the selectively-densified mesh and after the neural gas tuning. One can notice that a very good match is associated in both cases with the deformed area (shown in blue), but that the errors are lower in each case after neural gas tuning due to a better distribution of faces over the surface of the object. 

To quantify these results, we have also computed the Metro and perceptual errors for both the solutions proposed in [7] and the one proposed in this paper, as shown in Table 2 and Table 3, respectively. For the solution in our previous work, the overall perceptual similarity achieved is on average 74% over the entire surface of the object and roughly 91% over the deformed area, with an average computing time per object of 0.43 s on a Pentium III, 2 Ghz CPU, 64 bit operating system, 4 Ghz memory machine. 

The improvements in the current work, including the object alignment and a better selection of parameters, lead to an increase in performance, as can be noticed in Table 3. The overall perceptual similarity obtained is on average 86% (i.e., an improvement of 11.7% with respect to [7]) and 96.6% (i.e., an improvement of 5.9%) over the deformed area. The improved performance comes, however, at a higher average computation time per object, at an average of 0.70 s (roughly 39% higher with respect to the one reported in [7]). This change in computation time is mainly due to the alignment and realignment procedure and to the higher percentage of points chosen for the selectively-densified mesh construction.

Another series of tests is aimed at evaluating the performance of the solution for the prediction of the shape of an object using the series of feedforward neural networks, as described in Section 3.3.3. Networks are trained for 1000–2000 iterations, with an average training time of 15 s. The training error is of the order of e^−4^. Once trained, they can provide (in real-time) estimates for the position of the neural gas nodes. The mesh is reconstructed by the redistribution of faces using the Qslim algorithm, as detailed in Section 3.3.4. 

**Figure 10:**
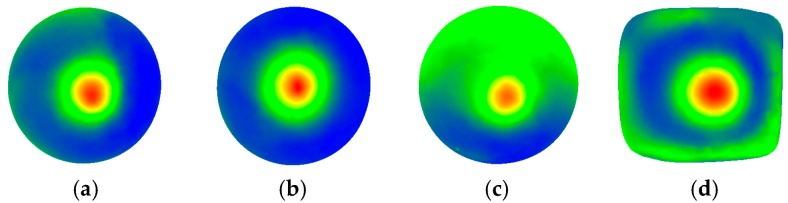
The color-coded difference between: (**a**) the ball model for *F_x_* = 3, *F_y_* = 17, *F_z_* = −226 and the predicted ball model for *F_x_* = 2, *F_y_* = 14, *F_z_* = −226, (**b**) the ball model for *F_x_* = −35, *F_y_* = 13, *F_z_* = −252 and the predicted ball model for: *F_x_* = −35, *F_y_* = 11, *F_z_* = −252, (**c**) the ball model for *F_x_* = −10, *F_y_* = 6, *F_z_* = −204 and the predicted ball model: *F_x_* = −10, *F_y_* = 5, *F_z_* = −200; and (**d**) the cube model and the predicted cube model.

Figure 10 shows a few examples of predicted deformed instances of objects obtained with the proposed solution, for the three objects under study. It is important to mention that we use here raw force data (i.e., $F_{x}$, $F_{y}$, and $F_{z}\;$components) as returned by the force-torque sensor, instead of the force magnitude computed in Equation (1), in order to enable the interpretation of the results. The objects are shown such that the deformation is situated along the *y* axis, and the *x* axis points towards the left side of the ball, while the *z* axis points towards the top of the image (as in Figure 4a). Studying Figure 10a that shows, for example, the difference between the ball model for *F_x_* = 3, *F_y_* = 17, and *F_z_* = −226 and the predicted ball model for *F_x_* = 2, *F_y_* = 14, and *F_z_* = −226, one can notice that there is a difference of force of 1 N along the *x* axis. Again, CloudCompare is used to encode in color the differences—blue representing a perfect match and the error increases from green, to yellow, orange, and red. The difference along the *x* axis is visible in the figure, in green, on the right side of the model. As one expects, the network was able to predict this movement of the object’s surface along the *x* axis.

Additionally, there is a difference between the forces applied on the *y* axis that is larger than the one over the *x* axis (i.e., 3 N). This difference is visible in green, red, and yellow around the deformation zone, as expected. For the case in Figure 10b, the force difference is mainly along the *y* axis and is reflected by differences in the deformation zone, as one might expect. A certain error appears around the sides of the object, as reflected by the green-bluish patches. The last example for the ball is for a force that affects the *z* and *y* directions and it is again correctly predicted. Finally, an example is shown for the estimation of the cube for a force varying with 4 N the along *y* axis. The difference shown in red, yellow, and green is mainly concentrated around this axis as well. 

To quantify the errors obtained for neural network training and prediction, we compute the Metro errors and the perceptual errors over the object surface and in the deformed area. The results for the training are shown in Table 4 and demonstrate that the network is able to capture quite accurately the neural gas nodes. One can notice that overall the errors for the ball are higher. This is mainly due to the fact that the ball that was used for experimentation is bigger in size than the cube and the sponge. Keeping the Kinect at roughly the same distance with respect to the probed object, the pointcloud collected for the ball contains more points than the cube and the sponge. For comparison, the number of points for the ball is 14,000, while for the cube and sponge it is 4680 and 5800, respectively. 

Table 5 quantifies the prediction errors when the networks are tested over previously unseen data, for 10 test cases, including those illustrated in Figure 10. It is important to notice that the differences in the force magnitude are not significant in the test scenarios with respect to the real measurements. The networks are only able to accurately predict within a limited region around the measured areas and for forces that do not differ significantly in magnitude and angle (i.e., about 4–5 N in force magnitude and 2–3° in angle). This is an important aspect that needs to be taken into consideration when designing solutions based on the training of neural networks for predicting deformable object behavior, and any data-driven solution in general. A large number of real data measurements are required to ensure a more accurate prediction. As expected, the errors are higher than those obtained for training, but still a good average similarity of 87.43% is preserved over the entire surface of an object and of roughly 96.6% in the deformed zone.

It is important to state as well that the complexity of the object’s geometry can also have an impact on the performance of the proposed framework. More complex shapes require more measurements than simpler objects in order to ensure a relatively accurate representation of the object’s behavior. This is due to the fact that when the force is applied to an arbitrary location, local deformation data might not be relevant for other regions where the geometric structure of the object (i.e., edges and corners) is different. One possible solution to this issue is to identify, based on visual information, the areas where the probing will take place such that the probing process is guided towards only relevant areas where changes in local geometry occur. While this topic is beyond the scope of this paper, a solution such as [24] could be used for this purpose. 

While a more complex geometry does not require any adjustment of the proposed framework, it would lead to an increase in the training time for the series of neural networks that map the interaction parameters with the local deformation, because of a larger volume of data to be processed. Due to the use of the neural gas and its ability to capture fine details in spite of a more complex shape, no additional challenges are expected at the level of simplification algorithm.

## 5. Conclusions

The paper proposes a novel solution to not only represent, but also predict the behavior of deformable 3D objects under interaction. Neural networks provide an efficient way to provide estimates in real-time while still using raw, noisy data, as opposed to the use of different approximations and assumptions on the object material, found nowadays in the literature. The proposed solution avoids recuperating elasticity parameters which cannot be precisely and accurately identified for certain materials such as foam, sponge, or rubber. The method can be used unaltered to characterize any object material, including even rigid objects, but it is expected that more measurements and therefore a longer training time are required for complex object shapes. In future work, a larger dataset of objects with various shapes and materials will be studied to better analyze the performance of the proposed framework. The framework will also be adapted and tested in the context of robotic manipulation tasks, in which the force-torque sensor measurements will be replaced by force measures exercised at the level of each robotic finger and the angle information by the position of each robotic finger.

## Figures and Tables

**Table 1:** Perceptual similarity measures according to neighborhood size, *n*.

	Ball	Cube	Sponge
	*n* = 8/*n* = 10/*n* = 12	*n* = 8/*n* = 10/*n* = 12	*n* = 8/*n* = 10/*n* = 12
Overall perceptual similarity (%)	71.38/71.38/71.38	79.3/78.87/78.5	83.75/83.37/82.81
Perceptual similarity (%) in the deformed zone	84.65/84.11/85.00	93.67/93.48/93.49	91.24/90.82/90.93

**Table 2:** Error measures and computing time for object representation with the solution in [7].

	Metro Overall Error (e^−3^)	Perceptual Overall Error (Similarity %)	Metro Error in Deformed Area (e^−5^)	Perceptual Error (Similarity%) in Deformed Area	Computing Time/Object
	Max/Mean/Rms		Max/Mean/Rms		
Ball	16.7/5.58/7.4	0.205 (79.5%)	24.3/3.05/4.69	0.082 (91.8%)	0.72 s
Cube	46.2/11.8/17.2	0.286 (71.4%)	25.7/3.47/5.07	0.127 (87.3%)	0.35 s
Sponge	21.6/5.09/7.55	0.281 (71.9%)	22.4/2.29/3.51	0.070 (93.0%)	0.23 s
Average	28.16/7.49/10.7	0.257 (74.3%)	24.13/2.93/4.42	0.093 (90.7%)	0.43 s

**Table 3:** Error measures and computing time for object representation with the current solution.

	Metro Overall Error (e^−3^)	Perceptual Overall Error (Similarity %)	Metro Error in Deformed Area (e^−5^)	Perceptual Error (Similarity%) in Deformed Area	Computing Time/Object
	Max/Mean/Rms		Max/Mean/Rms		
Ball	12.05/1.26/3.05	0.057 (94.30%)	8.84/0.63/2.43	0.001 (99.00%)	0.78 s
Cube	30.8/4.2/6.3	0.196 (80.40%)	22.5/2.10/5.01	0.028 (97.20%)	0.52 s
Sponge	21.3/2.2/3.3	0.171 (82.90%)	15.62/1.10/2.63	0.073 (92.70%)	0.80 s
Average	21.38/2.55/4.21	0.14 (86.00%)	15.65/1.27/3.36	0.034 (96.60%)	0.70 s

**Table 4:** Training error measures.

	Metro Overall Error (e^−3^)	Perceptual Overall Error (Similarity %)	Metro Error in Deformed Area (e^−5^)	Perceptual Error (Similarity%) in Deformed Area
	Max/Mean/Rms		Max/Mean/Rms	
Ball	17.10/3.30/2.70	0.201 (79.90%)	30.6/0.001/0.10	0.016 (98.40%)
Cube	5.40/0.13/0.56	0.060 (94.00%)	9.67/0.130/0.67	0.00016 (99.98%)
Sponge	2.60/0.63/0.77	0.0336 (96.64%)	2.70/0.001/0.09	0.0017 (99.83%)
Average	8.37/1.35/1.34	0.0982 (90.18%)	14.32/0.044/0.029	0.0060 (99.40%)

**Table 5:** Prediction error measures.

	Metro Overall Error (e^−3^)	Perceptual Overall Error (Similarity %)	Metro Error in Deformed Area (e^−5^)	Perceptual Error (Similarity%) in Deformed Area
	Max/Mean/Rms		Max/Mean/Rms	
Ball	20.1/3.00/4.20	0.236 (76.40%)	41.7/2.00/3.13	0.092 (90.80%)
Cube	5.40/0.18/0.70	0.078 (92.20%)	42.83/0.20/1.90	0.0006 (99.40%)
Sponge	2.60/0.65/0.80	0.063 (93.70%)	2.70/0.003/0.12	0.003 (99.70%)
Average	9.37/1.28/1.90	0.1257 (87.43%)	29.07/0.73/1.72	0.032 (96.63%)
